# Role of Plasminogen Activator Inhibitor Type 1 in Pathologies of Female Reproductive Diseases

**DOI:** 10.3390/ijms18081651

**Published:** 2017-07-29

**Authors:** Yao Ye, Aurelia Vattai, Xi Zhang, Junyan Zhu, Christian J. Thaler, Sven Mahner, Udo Jeschke, Viktoria von Schönfeldt

**Affiliations:** Department of Gynaecology and Obstetrics, Ludwig-Maximilians University of Munich, Campus Großhadern: Marchioninistr. 15, 81377 Munich and Campus Innenstadt: Maistr. 11, 80337 Munich, Germany; Yao.Ye@med.uni-muenchen.de (Y.Y.); Aurelia.Vattai@med.uni-muenchen.de (A.V.); Xi.Zhang@med.uni-muenchen.de (X.Z.); Junyan.Zhu@med.uni-muenchen.de (J.Z.); Thaler@med.uni-muenchen.de (C.J.T.); Sven.Mahner@med.uni-muenchen.de (S.M.); Viktoria.Schoenfeldt@med.uni-muenchen.de (V.v.S.)

**Keywords:** plasminogen activator inhibitor type 1, trophoblast invasion, recurrent pregnancy losses, preeclampsia, intrauterine growth restriction, gestational diabetes mellitus, endometriosis, polycystic ovary syndrome

## Abstract

Normal pregnancy is a state of hypercoagulability with diminishing fibrinolytic activity, which is mainly caused by an increase of plasminogen activator inhibitor type 1 (PAI-1). PAI-1 is the main inhibitor of plasminogen activators, including tissue-type plasminogen activator (tPA) and urokinase-type plasminogen activator (uPA). In human placentas, PAI-1 is expressed in extravillous interstitial trophoblasts and vascular trophoblasts. During implantation and placentation, PAI-1 is responsible for inhibiting extra cellular matrix (ECM) degradation, thereby causing an inhibition of trophoblasts invasion. In the present study, we have reviewed the literature of various reproductive diseases where PAI-1 plays a role. PAI-1 levels are increased in patients with recurrent pregnancy losses (RPL), preeclampsia, intrauterine growth restriction (IUGR), gestational diabetes mellitus (GDM) in the previous pregnancy, endometriosis and polycystic ovary syndrome (PCOS). In general, an increased expression of PAI-1 in the blood is associated with an increased risk for infertility and a worse pregnancy outcome. GDM and PCOS are related to the genetic role of the 4G/5G polymorphism of *PAI-1*. This review provides an overview of the current knowledge of the role of PAI-1 in reproductive diseases. PAI-1 represents a promising monitoring biomarker for reproductive diseases and may be a treatment target in the near future.

## 1. Introduction

The fibrinolytic system plays a role in several physiological and pathophysiological processes, such as hemostatic balance, tissue remodeling, tumor invasion, angiogenesis and reproduction [1]. Normal pregnancy is a state of hypercoagulability with remarkable changes in all aspects of hemostasis—an increase of clotting factors and coagulability and a decrease of anticoagulants and fibrinolytic activity, thereby influencing placental function during pregnancy and meeting delivery’s hemostatic challenge [2]. Fibrinolytic system is depressed during pregnancy, and this change partly explains the higher incidence of thromboembolic complications such as recurrent pregnancy losses, preeclampsia and intrauterine growth restriction [2]. The diminishing fibrinolytic activity is mainly caused by a continuous increase of the major inhibitor of the fibrinolytic system: plasminogen activator inhibitor type 1 (PAI-1) [2]. PAI-1 is responsible for approximately 60% of the PA-inhibitory activity in the plasma [3] and is the key inhibitor of fibrinolysis compared with PAI-2 and PAI-3 during pregnancy [4].

*PAI-1* gene deficiency shows a transient impaired placentation in mice [5], while, in humans, *PAI-1* gene deficiency is associated with abnormal bleeding after a trauma or surgery [6,7]. Transgenic mice that overexpress PAI-1 exhibit thrombotic occlusion [8]. Former studies in humans suggest that increased PAI-1 levels are found to be crucial mediators of vascular disease, fibrosis, tumor metastasis, diabetes, and reproductive diseases [9,10,11,12]. PAI-1 acts as a major inhibitor of fibrinolysis, its overexpression leads to fibrin accumulation and insufficient placentation. In this review, we focus on the complex roles of PAI-1 in normal placentation and reproductive diseases, including recurrent pregnancy losses, preeclampsia, intrauterine growth restriction, endometriosis and polycystic ovary syndrome.

## 2. Fibrinolytic System and PAI-1 (Plasminogen Activator Inhibitor Type 1)

**Figure 1:**
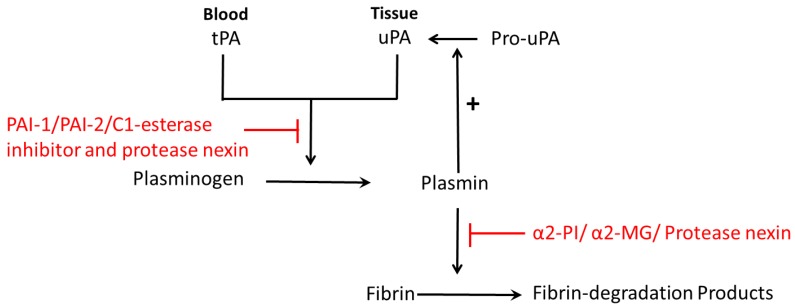
Schematic diagram of fibrinolysis: plasminogen is activated by plasminogen activator (tPA in blood or uPA in tissue), and then converted to plasmin. Then plasmin cleaves fibrin into fibrin-degradation products. Plasminogen activators inhibitors are PAI-1, PAI-2, C1-esterase inhibitor and protease nexin. Plasmin inhibitors are α2-plasmin inhibitor (α2-PI), α2-macroglobulin (α2-MG) and protease nexin. Pro-uPA can be converted to uPA, which is catalyzed by plasmin, the product of plasminogen.

The prime fibrinolytic protease of the fibrinolytic system is plasminogen, which can be activated by urokinase-type plasminogen activator (uPA) and the tissue-type plasminogen activator (tPA) [1,8]. Plasminogen can then be converted into plasmin, and eventually cleaves fibrin into cross-linked fibrin degradation products (Figure 1) [8]. Plasminogen activator inhibitors include PAI-1, PAI-2, PAI-3, C1-esterase inhibitor and protease nexin (Figure 1) [8]. Plasmin inhibitors are α2-plasmin inhibitor (α2-PI), α2-macroglobulin (α2-MG) and protease nexin (Figure 1) [8]. Both uPA and tPA are serine proteases that cleave a single Arg-Val peptide bond to transfer plasminogen to plasmin; uPA functions mainly in pericellular proteolysis while tPA is involved in the circulation [13]. uPA plays an important role in a variety of physiological and pathological processes including tissue destruction, inflammatory reactions and invasion of trophoblasts [14] and cancer cells [15]. Both uPA and tPA consist of a single-chain form and a two-chain form [4,16]. During normal pregnancy, the levels of uPA, PAI-1, PAI-2 and α2-antiplasmin are increased and tPA levels are decreased [17].

PAI-1 is the primary inhibitor of tPA in the plasma during pregnancy [4]. It is a single-chain glycoprotein consisting of 379 or 381 amino acids (N-terminal heterogeneity) and belongs to the serine family of protease inhibitor, with a molecular weight of about 45 kDa. There are three different forms of PAI-1: active, inactive and substrate form. The active form can inhibit tPA or uPA by forming a 1:1 stoichiometric complex with each enzyme and the inactive form does not react with the target proteinase [18]. The conformational conversion from the active into the inactive form is completed by the P1-P1’ in a reactive center loop (RCL) of the serpin cleave, followed by the insertion of the RCL into the β-sheet A of the serpin [19]. *PAI-1* gene in humans is located on chromosome 7 (q21.3-q22), extends approximately 12.200 base pairs and consists of nine exons and eight introns [20]*. PAI-1* gene has several polymorphisms and the 4G allele of the 4G/5G polymorphism is related to high PAI-1 levels [21]. 4G polymorphism is located in the PAI-1 promotor, which is 675 bp upstream from the start site of transcription in the promoter region [21]. Circulating PAI-1 is mainly found in platelets, whilst a large range of cells can further express PAI-1, such as fibroblasts, smooth muscle cells, endothelial cells, hepatocytes, inflammatory cells and placental cells [22].

Both forms of tPA are inhibited by PAI-1, whereas PAI-2 inhibits mainly the two-chain form [23]. PAI-2 consists of two molecular forms: the low molecular weight (LMW) form with 43–48 kDa is intracellular and non-glycosylated, while the high molecular weight (HMW) form with 60 kDa is secreted and glycosylated [24]. PAI-2 is mainly expressed by placental trophoblasts and macrophages [23]. PAI-3, also known as protein C inhibitor (PCI), is prominently expressed in male reproductive organs and its low levels in seminal plasma are associated with infertility [25].

## 3. Role of PAI-1 in the Female Reproduction System

During a healthy pregnancy, PAI-1 levels in the plasma gradually elevate during the second trimester of pregnancy and reach a maximum at 32–40 weeks of pregnancy. Within 5–8 weeks after delivery, PAI-1 levels fall again to the levels before the occurrence of pregnancy [2]. The concentration of PAI-1 in the plasma of healthy non-pregnant women varies (55 ± 17 ng/mL) [26]. The maximum of PAI-1 concentration during the last trimester of pregnancy is approximately 3–5 times higher than that of non-pregnant women [26]. These temporary changes of PAI-1 during pregnancy are accounted for hormonal influences [27]. Increases in PAI-2 levels are observed during pregnancy and during delivery [23]. Both PAI-1 and PAI-2 decrease quickly following the placenta separation from the uterus, but PAI-2 can still be found in the circulation up to eight weeks postpartum [2].

Trophoblasts can express PAI-1 and PAI-2 in vivo and in vitro [28]. Both PAI-1 and PAI-2 are localized in the cytoplasm of cytotrophoblasts and in the cytoplasm and plasma membrane of intermediate and syncytiotrophoblasts [28]. PAI-1 is localized in invading trophoblasts in the human placenta by immunostaining [29], especially in extravillous interstitial trophoblasts and vascular trophoblasts [30]. In the human placenta, PAI-1 protein and mRNA expression exist in most extravillous cytotrophoblast cells of the decidual layer, especially the chorionic villous tree and in cytotrophoblast cells of the chorionic plate, basal plate and intercotyledonary septae [31]. No expression of PAI-1 has been observed in the basal plate of endometrial stromal cells, chorionic plate connective tissue cells, septal endometrial stromal cells or villous core mesenchyme [31]. PAI-2 is the predominant PAI accumulated in villous syncytiotrophoblasts [29]. Both PAI-1 and PAI-2 mRNAs expressions are detected in cultured cytotrophoblasts isolated from both the first trimester and term placenta [29]. PAI-1 and PAI-2 are also expressed by uterine natural killer (uNK) cells [32].

### 3.1. PAI-1 Inhibits Trophoblast Invasion

**Figure 2:**
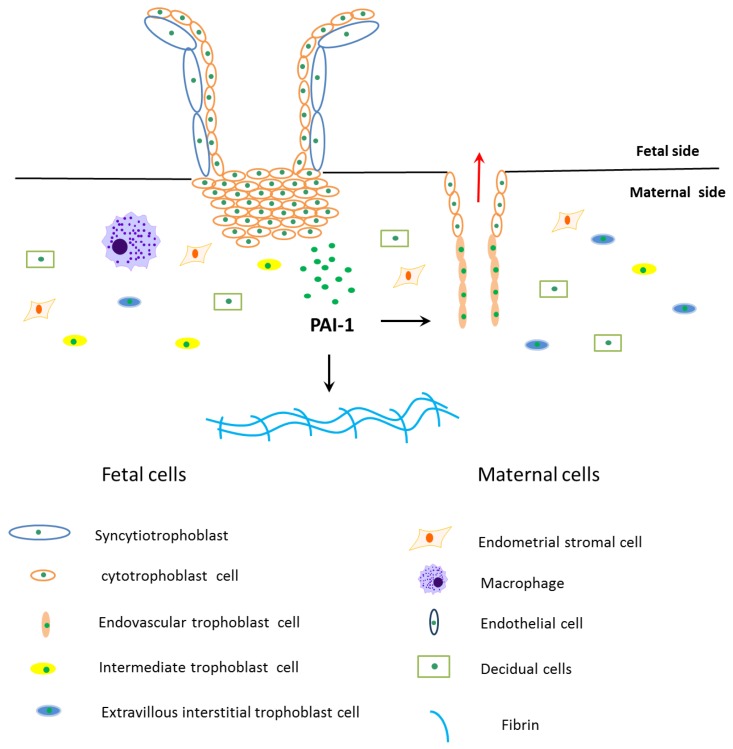
Schematic diagram of PAI-1’s role during trophoblasts invasion. The fetal side of the human placenta mainly includes cytotrophoblasts and syncytiotrophoblasts, and sycytiotrophoblasts are differentiated and fused by cytotrophoblasts. The cytotrophoblasts invade into the maternal side and differentiate into extravillous interstitial trophoblasts, intermediate trophoblasts and endovascular trophoblasts. Among them, extravillous interstitial trophoblasts and endovascular trophoblasts express plasminogen activator inhibitor type 1 (PAI-1). Furthermore, cells from the maternal side take part in trophoblast invasion, such as endometrial stromal cells, decidual cells, macrophages and endothelial cells. Extravillous trophoblast invasion in early pregnancy is precisely controlled by many factors expressed by trophoblasts and maternal cells, where PAI-1 is the main anti-invasive factor. PAI-1 prevents trophoblast invasion by inhibiting extracellular matrix degradation, which leads to fibrin accumulation in the maternal side. PAI-1 may also play a role in remodeling maternal uterine spiral arteries.

Trophoblast invasion at the maternal-fetal interface is a key process during implantation and placentation, and during this process extravillous cytotrophoblasts (EVT) acquire invasive properties, which are able to invade and remodel maternal tissues (interstitial EVT) and uterine spiral artery (endovascular EVT) [33]. EVT can degrade extracellular matrix (ECM) to promote cell migration to the maternal side [33]. This process is precisely controlled by many factors expressed by maternal cells and trophoblasts (Figure 2) [33].

Trophoblasts and malignant tumors use the same biochemical mediators to facilitate invasion, including extracellular matrix degradation and immunosuppression of environmental conditions. PAI-1 can inhibit trophoblasts invasion while promoting tumor cell immigration [34,35]. PAI-1 is a biomarker for malignancies with poor prognosis because it facilitates tumor cell migration and invasion [35]. PAI-1/uPA/uPA receptor (uPAR)/low density lipoprotein receptor-related protein (LRP)/integrin complexes are initiating an “adhesion–detachment–re-adhesion” cycle to promote tumor cell migration [35,36]. Hyperinvasiveness in premalignant and malignant extravillous trophoblasts (JAR and JEG-3 choriocarcinoma cell lines) results from a downregulation of tissue inhibitors of metalloprotease (TIMP)-1 and *PAI-1* genes [34].

EVT invasion in early pregnancy occurs in a relatively low-oxygen (3%) environment, which is mediated by a general inhibition of the plasminogen activator system [37], as well as many adhesion molecules, growth factors, cytokines, interleukins, ECM components, and various placental hormones [14,33]. The anti-invasive action of EVT is caused by an upregulation of the tissue inhibitor of TIMP-1 and PAI-1 and a downregulation of uPA [14]. PAI-1 and PAI-2 are expressed in invading human extravillous trophoblast cells and they limit the depth of invasion [37,38]. PAI-1 is found to be absent in the placental bed of ectopic and molar pregnancies, suggesting that no expression of PAI-1 can contribute to an uncontrolled placental invasion [30]. Expression of PAI-1 is elevated in the process of EVT invasion treated by tumor necrosis factor α (TNF-α), and adding PAI-1-inactivating antibodies restores migration [39].

The limitation of EVT invasion is due to reduced ECM degradation (Figure 2), which requires the balance of promoting and restraining factors, such as metalloproteinases (MMPs) and tissue inhibitors of MMPs (TIMPs), uPA and PAI-1 [40]. PAI-1 restrains ECM degradation in three different ways. Firstly, PAI-1 can directly block uPA activity to constrain the proteolytic activity of plasmin [18]. Secondly, PAI-1 binds to uPA and causes its degradation via uPAR and low-density lipoprotein receptor-related protein (LRP) internalization [41]. The direct binding of occupied uPAR to LRP is essential for internalization and clearance of uPA-PAI-1-occupied uPAR [42]. The activity of PAI-1 depends on its interactions with LRP, which leads to the activation of the Janus kinase 2/signal transducer and activator of transcription protein (Jak/-Stat) signaling pathway [43]. Lastly, an increase in the *PAI-1* transcript and translation leads to the formation of keloids and fibrosis [44]. Renaud et al. (2005) suggested that macrophages participate in decreasing the depth of trophoblast invasion by secreting TNF-α, which couples TNFR and promotes EVT to release PAI-1 during placentation [45]. Huber et al. (2006) further reported that TNF-α stimulates PAI-1 level of HTR-8/SVneo cells by activation of the nuclear factor κ-light-chain-enhancer of activated B cells (NF-κB) pathway [46].

PAI-1 may also play a role in remodeling maternal uterine spiral arteries (Figure 2) [47]. PAI-1 mRNA positive cells in the maternal arteriole co-express cytokeratin, implying that PAI-1 may participate in the process of endovascular cytotrophoblast cells replacing cells of the arteriole wall [47]. Hypoinvasion and failed placental vascular remodeling are associated with reproductive diseases, such as recurrent pregnancy losses (RPL), preeclampsia and intrauterine growth restriction (IUGR) and even maternal as well as fetal death [48,49]. In terms of tumor cells, PAI-1 can both promote and inhibit tumor growth and angiogenesis. Low concentrations of PAI-1 can stimulate tumor angiogenesis while treatment of animals with high doses of PAI-1 suppresses angiogenesis and tumor growth [50].

### 3.2. Recurrent Pregnancy Losses

Recurrent pregnancy losses (RPL) affects approximately 1% of all couples worldwide [51], and its latest definition is two or more consecutive failed pregnancies as documented by ultrasonography or histopathologic examination before the 20th pregnancy week according to the Practice Committee of the American Society for Reproductive Medicine [52]. The identifiable reasons for RPL include genetic abnormalities, structural abnormalities, infection, endocrine abnormalities, immune dysfunction, and thrombophilic disorders [53]. However, there are still up to 40–50% of pregnancy losses that have unidentifiable causes [54]. PAI-1 plasma levels in RPL patients are increased in comparison to women with healthy pregnancies [55]. Among all the thrombophilic genes, functional *PAI-1*-675 4G/5G polymorphism is one of the most frequently analyzed PAI-1 genetic variants. However, the contribution of *PAI-1*-675 4G/5G to unexplained RPL has remained controversial.

Gris et al. (1997) analyzed a study with 500 women with unexplained RPL and demonstrated that increased PAI-1 levels are the most frequent hemostasis-related abnormality connected to unexplained RPL [56]. A recent meta-analysis by Li et al. (2015) including 22 studies with 4306 cases and 3076 controls showed that *PAI-1* 4G/5G polymorphism is associated with an increased RPL risk (*p* = 0.0003), especially in the Caucasian subgroup (*p* < 0.001) [57]. Khosravi et al. (2014) further found that a high prevalence of *PAI-1* -675 4G/4G existed in RPL patients as well as in implantation failure (IF) patients (*p* < 0.001) [58].

Another group suggests that *PAI-1*-675 4G/5G alone is not responsible for RPL [59,60], and more thrombophilic gene mutations together can help predispose RPL risk. More than three gene mutations among the 10 thrombophilic gene mutations (*factor V* G1691A, *factor V* H1299R (R2), *factor V* Y1702C, *factor II pro-thrombin* G20210A, *factor XIII* V34L, *b-fibrinogen* −455G > A, *PAI-1* 4G/5G, *HPA1* a/b (L33P), *MTHFR* C677T, and *MTHFR* A1298C) are more prevalent in RPL patients [61,62]. Endothelial PAI-1 synthesis is induced by angiotensin II, which is generated by angiotensin I-converting enzyme (ACE) and this might be one of the main reasons of elevated PAI-1 concentrations in RPL [63].

In general, associations between *PAI-1* 4G/5G and unexplained RPL may have been masked by sample-size effects, ethnicity, enrollment criteria or combinations. In order to improve our understanding of the pathobiology of RPL, we need to identify not only novel genetic variants and the interaction, but also how genes, proteins and the environment contribute to RPL. Thus far, PAI-1 inhibiting fibrinolysis and fibrin accumulation are believed to be the principle reasons for RPL, but the understanding of the mechanism of PAI-1 in RPL still has to be further analyzed.

### 3.3. Preeclampsia

Preeclampsia affects about 2.5% to 3.0% of pregnant women [64] and it is a leading cause of perinatal morbidity and mortality both for the fetus and the mother [65]. Preeclampsia is a pregnancy disorder characterized by hypertension, proteinuria and placental abnormalities [66], which are generally manifested in the second to third trimester of pregnancy [67]. Although the mechanisms responsible for the pathogenesis of preeclampsia are poorly understood, there is an agreement that it is associated with hypoinvasion and failed conversion of maternal endometrial spiral arteries in the placenta [64], both of which are related to PAI-1 and PAI-2.

Maternal PAI-1 levels in the plasma are higher in patients with preeclampsia during the second trimester of pregnancy [67], and its mRNA levels are positively correlated with the severity of preeclampsia during 35–41 weeks of gestation [68]. The ratio of PAI-1 to PAI-2 is increased in women with early-onset preeclampsia (24–32 gestational weeks) in comparison to the control group, but not in late-onset preeclampsia (35–42 gestational weeks) [69]. Estelles et al. (1989) found that both antigenic and functional PAI-1 levels are increased while antigenic and functional levels of PAI-2 are decreased in the third trimester (≥28 weeks) compared with healthy controls [70]. In contrast, in the first trimester (11–13 weeks), PAI-2 in the plasma is observed to be not differently expressed in preeclampsia [71]. PAI-1 antigen levels are positively correlated to proteinuria in women with preeclampsia [72]. Therefore, PAI-1 has been considered as a potentially useful predictor of preeclampsia. Although it is known that smoking may reduce the risk for preeclampsia [73], specifically chronic smoking upregulates PAI-1 levels [74].

It remains uncertain whether increased PAI levels are the primary mechanism leading to preeclampsia or a consequence of the associated endothelial and placental damage [75]. Cytotrophoblasts, which do not express Raf kinase inhibitor protein (RKIP), could be one of the reasons for impaired migration of cytotrophoblasts in preeclampsia, because locostatin (the inhibitor of RKIP) induces PAI-1 expression with the support of activation of NF-κB pathway and finally contributes to an inadequate trophoblast invasion [76].

PAI-1 expression in the plasma is increased following exposure to inflammatory cytokines, including interleukin 1β (IL-1β) [77], vascular endothelial growth factor (VEGF), epidermal growth factor (EGF) and fibroblast growth factor (FGF) or hypoxic conditions [78]. Hypoxia can directly stimulate PAI-1 mRNA and protein expression [35], and can also stimulate hypoxia-inducible transcription factors (HIF-1α and HIF-2α) to induce PAI-1 [79], both of which may also be the mechanisms of preeclampsia. When preeclampsia occurs in combination with increased levels of syncytial PAI-1, intervillous fibrin deposition and infarction may reduce the flow of nutrients from mother to fetus leading to IUGR [80].

Still, there is a dispute of the correlation between *PAI-1* polymorphism and preeclampsia. Gerhardt et al. (2005) discovered that women with *PAI-1* 5G/5G genotype are at risk for early onset of severe preeclampsia (17–35 gestational weeks) [81]. Morgan JA et al. (2013) found that *PAI-1-*675 4G/5G polymorphism is not associated with preeclampsia with a total of 5003 women involved in the meta-analysis [82].

### 3.4. Intrauterine Growth Restriction

Intrauterine growth restriction (IUGR) is defined as fetuses with pathological smallness caused by an underlying functional problem [83], occurring in 5–10% of all pregnancies [84]. IUGR is associated with increased risks of neonatal death or disability in the perinatal period [84,85] and predisposes the child to a lifelong increased risk for hypertension, cardiovascular disorders and renal disease [86]. Histopathological studies of IUGR placentas indicate abnormalities of the maternal spiral arterioles, dysregulated villous vasculogenesis, and abundant fibrin deposition, as well as oxidative stress and apoptosis in villous trophoblast [87].

PAI-1 is a potential marker of placental insufficiency and it is associated with fetal hypoxia and angiogenesis in IUGR [84]. PAI-1 levels in the umbilical cord blood are increased in patients with IUGR and it is associated with the plasma’s angiogenic potency measured in vitro [84]. Cytotrophoblast cells isolated from the placenta of IUGR pregnancies express significantly higher levels of PAI-1, with a significant decrease in plasminogen activator activity, compared with trophoblast cells from normal pregnancy cultured in vitro [88]. This localized increased production of PAI-1 may play an important part in restricting endovascular trophoblast invasion in early pregnancy, increasing fibrin deposition and reducing uteroplacental blood flow in IUGR pregnancies [88].

PAI-1 placental levels are increased in pregnancies with IUGR and preeclampsia, but not in patients with isolated IUGR [89]. In contrast, PAI-2 expression is reduced in placentas of both IUGR women with and without preeclampsia compared with normal placentas [89]. In the first trimester, low PAI-2 is associated with a higher risk for the development of IUGR [90]. A significant decrease in PAI-2 in the plasma and amniotic fluid is observed in IUGR groups in comparison with normal pregnancies [91]. PAI-2 levels are correlated with fetal weight of IUGR pregnancies [92,93], indicating that PAI-2 is not only the marker of the quantity and quality of the placenta tissues but also a marker for fetal growth and development [17]. *PAI-1* polymorphism (4G/5G) has not been associated with an increased risk of IUGR in the case-control analysis [94,95].

### 3.5. Gestational Diabetes Mellitus

Gestational diabetes mellitus (GDM) is characterized by impaired glucose tolerance during pregnancy, with the prevalence of 0.6–20% of pregnancies [96]. Women with GDM can have complications including polyhydramnios, fetal macrosomia, preeclampsia, shoulder dystocia and increased risk for operative delivery. Postpartum, patients with GDM have a more than seven-fold increased risk of developing postpartum diabetes compared to women without GDM [97]. Insulin resistance and chronic subclinical inflammation are the two main pathways leading to GDM and possibly previous GDM [98]. Insulin resistance syndrome is associated with increased levels of leptin, TNF-α, tPA, PAI-1 and testosterone [99].

PAI-1 is partly secreted by adipose tissue, and can lead to an impairment of the fibrinolytic system [100]. Salmi et al. (2012) reported that PAI-1 levels are higher in the serum of women with GDM compared to healthy women during the early third trimester pregnancy [101]. Bugatto et al. (2017) stated that PAI-1 levels in maternal uterine blood do not change in women with GDM at the third trimester pregnancy compared to controls [102]. McManus et al. (2014) suggested that GDM women have lower concentrations of PAI-1 in comparison to the age- and weight-matched controls in maternal plasma, as well as in the umbilical artery and umbilical vein [103]. This study further demonstrated that GDM offspring also have decreased PAI-1 concentrations compared to controls [103].

PAI-1 levels in women with GDM are not consistent, but PAI-1 levels have been shown to be increased in women who had GDM during a previous pregnancy [98]. Women with previous GDM have elevated PAI-1 levels 0.25–4 years after delivery [98]. PAI-1 levels are correlated with the insulin sensitivity index (SI), and the PAI-1/SI ratio is increased in women with a previous GDM and impaired insulin [104].

PAI-1 expression is significantly correlated with a variety of adiposity features, including body mass index (BMI), total fat mass, waist circumference, visceral adipose tissue and subcutaneous adipose tissue, total cholesterol triglycerides, fasting plasma glucose and 2 h plasma glucose in the glucose tolerance test, insulin sensitivity as well as pancreatic beta-cell function [100,105,106]. Hyperglycemia inhibits the expression of uPA and PAI-1 by the induction of p38 mitogen-activated protein kinases (p38 MAPK) and peroxisome proliferator-activated receptor γ (PPAR-γ) stress signaling pathways, which are different from the PAI-1 levels induced during preeclampsia [107].

Leipold et al. (2006) reported that the 5G allele of the *PAI-1* gene is associated with normal glucose tolerance in pregnant women independent of maternal age or BMI and it is also related to low fasting glucose values in the oral glucose tolerance test [108]. Physical activities can lower cardiometabolic risk in women with previous GDM and PAI-1 levels, as well as the level of C-reactive protein, leptin and triglycerides [109].

Furthermore, Glueck et al. (2000) found that the polymorphism of *PAI-1* gene is not associated with eclampsia, abruptio placentae and intrauterine fetal death by stepwise logistic regression [110].

### 3.6. Endometriosis

Endometriosis is defined as the presence of endometrial-like tissue outside the uterus, causing dysmenorrhea, chronic pelvic pain, dyspareunia, and infertility [111]. Pathogenesis of endometriosis includes inflammation, angiogenesis, cytokine/chemokine expression and endocrine alterations such as estrogen receptors (ESRs) and progesterone receptors (PGRs) expression [111]. PAI-1 plays an important role in tumor cell migration and invasion [11], which is also the mechanism of endometriotic cells invasion [112].

Ovarian endometriotic tissues have higher antigenic levels of PAI-1 than normal endometrium [113]. Increased PAI-1 antigen levels in peritoneal fluid from patients with endometriosis contribute to an increase of peritoneal adhesions [114]. In vitro, isolated endometriotic stromal cells release a higher level of PAI-1 compared to the endometrial stromal and epithelial cells of women with endometriosis and controls [112].

There exists a dispute of the association between *PAI-1* gene polymorphism 4G/5G and endometriosis [115,116]. *PAI-1* 4G/5G polymorphism is associated with an increased risk for endometriosis-associated infertility [116]. However, Goodarzi et al. found that *PAI-1* genotype distribution is similar in patients with endometriosis and controls [115].

### 3.7. Polycystic Ovary Syndrome

Polycystic ovary syndrome (PCOS) is the most common endocrinopathy of women in the reproductive age, with a prevalence of up to 10% and symptoms include hyperandrogenism and/or hyperandrogenemia, oligo-ovulation and polycystic ovarian morphology [117]. PCOS is associated with infertility, RPL, type 2 diabetes mellitus and cardiovascular diseases [117]. Many studies in women with PCOS have reported that PAI-1 levels in the plasma are increased in both normal weight and overweight/obese women with PCOS compared with controls with matched body mass index (BMI) [100,118,119]. Serum PAI-1 activity is related to the BMI and homeostasis model assessment (HOMA) score [120], insulin levels and insulin sensitivity indices [121]. PAI-1 levels decline after treatment with sibutramine and metformin in normal weight and overweight women with PCOS [118,122].

Oligo-ovulation (defined as delayed menses >35 days or <8 spontaneous hemorrhagic episodes per year) is not completely understood yet [123]. uPA plays an essential role in the early growing follicles during cell proliferation and migration, and in the early corpus luteum (CL) formation related to ECM degradation and angiogenesis [124]. PAI-1 is localized in the granulosa and theca cells, indicating it possibly plays a role in human ovulation, but its role in PCOS needs to be further explained [125]. Increased PAI-1 expression in CL of rat and monkey at a later stage is correlated with a sharp decrease in progesterone production of CL [124]. The role of PAI-1 in ovulation needs to be studied further in humans. PAI-1 is a predominant independent risk factor for miscarriages in women with PCOS [126]. Elevated plasma PAI-1 levels are associated with an increased risk for both type 2 diabetes mellitus and cardiovascular diseases (i.e., coronary disease/ischemic stroke) [127,128,129].

*PAI-1* 4G/5G polymorphism is associated with susceptibility to PCOS in European, Turkish, and Asian populations [130,131].

## 4. Conclusions

PAI-1 is expressed in the placenta and maternal plasma of pregnant women. In the human placenta, PAI-1 is localized in invading trophoblasts, especially in extravillous trophoblasts. By inhibiting ECM degradation, PAI-1 plays a vital role in the prevention of trophoblast invasion in RPL, preeclampsia and IUGR. Increased expression of PAI-1 in the plasma is found in RPL, preeclampsia, IUGR, GDM in previous pregnancies, endometriosis and PCOS. Similar to tumor cells, PAI-1 promotes endometriotic cells invasion during endometriosis. In PCOS, PAI-1 may regulate ovulation but this hypothesis still requires further research. *PAI-1* 4G/5G polymorphism is associated with GDM and PCOS; however, contradictory results are found in patients with RPL, preeclampsia and endometriosis. PAI-1 expression can be reduced through physical exercise and pharmacological interventions, such as the oral intake of thiazolidinedines (troglitazone or pioglitazone) [100], statins [100] and metformin [118]. Altered PAI-1 levels have further been found in other pathologies. Studies on cardiovascular diseases have displayed a strong positive correlation between PAI-1 levels in the serum and cardiovascular risks for myocardial infarction (MI), recurrent MI, angina pectoris and atherosclerosis [9]. In addition, PAI-1 is a pivotal mediator of vascular diseases, cancer, asthma, insulin resistance and diabetes [100]. In the future, PAI-1 may function as a biological monitoring parameter and possibly represents a therapeutic approach in reproductive pathologies.

## Abbreviations

PAI	plasminogen activator inhibitor
tPA	tissue-type plasminogen activator
uPA	urokinase-type plasminogen activator
ECM	extra cellular matrix
α2-PI	α2-plasmin inhibitor
α2-MG	α2-macroglobulin
RCL	reactive center loop
LMW	low molecular weight
HMW	high molecular weight
PCI	protein C inhibitor
uNK	uterine natural killer
EVT	extravillous cytotrophoblast
uPAR	uPA receptor
LRP	low density lipoprotein receptor-related protein
TIMP	tissue inhibitors of metalloprotease
TNF-α	tumor necrosis factor α
MMP	metalloproteinase
Jak/-Stat	Janus kinase 2/signal transducer and activator of transcription protein
NF-κB	nuclear factor κ-light-chain-enhancer of activated B cells
ACE	angiotensin I-converting enzyme
IL-1β	interleukin 1β
VEGF	vascular endothelial growth factor
EGF	epidermal growth factor
FGF	fibroblast growth factor
HIF	hypoxia-inducible transcription factors
p38 MAPK	p38 mitogen-activated protein kinases
PPAR-γ	peroxisome proliferator-activated receptor γ
RKIP	raf kinase inhibitor protein
ESR	estrogen receptor
PGR	progesterone receptor
SI	sensitivity index
BMI	body mass index
HOMA	homeostasis model assessment
CL	corpus luteum
RPL	recurrent pregnancy losses
IF	implantation failure
IUGR	intrauterine growth restriction
GDM	gestational diabetes mellitus
PCOS	polycystic ovary syndrome
MI	myocardial infarction
